# A seven-gene diagnostic and prognostic signature for papillary thyroid carcinoma

**DOI:** 10.1007/s12672-026-05473-4

**Published:** 2026-06-21

**Authors:** Berna Hukkamlı, Burak Dagdelen, Feyza Sönmez Aydın

**Affiliations:** 1 Department of Chemical and Chemical Processing Technologies, Boyabat Vocational School, Sinop University, Sinop, 57200 Türkiye; 2 Department of Medical Biology, Faculty of Medicine, Selcuk University, Konya, 42130 Türkiye; 3 Department of Molecular Biology and Genetics, Science Faculty, Atatürk University, Erzurum, 25240 Türkiye

**Keywords:** Papillary thyroid carcinoma, Gene expression signature, LASSO regression, DNA methylation, Immune infiltration

## Abstract

Papillary thyroid carcinoma (PTC) is the most common subtype of thyroid cancer; however, indeterminate cytology in fine-needle aspiration biopsy (FNAB) and the limited sensitivity of single–mutation–based biomarkers remain important clinical challenges. This study aimed to identify a robust multi-gene expression signature for diagnostic evaluation and exploratory prognostic assessment of PTC. An integrative bioinformatics framework was applied to TCGA-THCA RNA-sequencing data (505 tumors and 59 normal samples). Differentially expressed genes were subjected to LASSO regression to construct a diagnostic model, which was externally validated in two independent GEO cohorts (GSE33630 and GSE60542). A seven-gene signature consisting of four upregulated genes (*GABRB2*, *SERINC2*, *PTCHD4*, *HAPLN1*) and three downregulated genes (*SLC6A15*, *UGT2B11*, *GRIA1*) demonstrated excellent diagnostic performance in the discovery cohort (AUC = 0.995) and remained highly accurate in external validation datasets (AUC = 0.940 and 0.938). Kaplan–Meier analysis showed significant stratification for disease-free interval (p = 0.034), although this association did not remain independent in multivariable analysis. Exploratory analyses suggested potential epigenetic regulation of several genes and distinct patterns in immune microenvironments between risk groups. Overall, these findings reveal a robust seven-gene expression signature with strong diagnostic capacity and potential prognostic relevance in PTC, highlighting its value for molecular characterization. However, further biological validation and prospective evaluation in clinically relevant cohorts are required before translational applicability can be established.

## Introduction

Thyroid cancer is the most common malignancy of the endocrine system, and its rapidly increasing global incidence has attracted considerable attention in recent years. According to data from the International Agency for Research on Cancer [1], thyroid cancer ranks as the ninth most common malignancy worldwide and the fifth most frequently diagnosed cancer among women, with approximately 586,000 new cases and 44,000 deaths reported in 2020 [2]. Thyroid cancer is considered a multifactorial disease arising from complex interactions among environmental exposures—such as heavy metals and radiation—hormonal influences, and genetic and molecular drivers [3]. Histopathologically, thyroid cancers are classified into papillary, follicular, anaplastic, and medullary subtypes, with papillary thyroid carcinoma accounting for approximately 85–90% of all cases [4].

Papillary thyroid carcinoma (PTC) generally has a favorable prognosis, with a 5-year survival rate of approximately 98% in early-stage disease [5]. However, the risk of recurrence persists in approximately 5–21% of patients [6], and survival declines dramatically to nearly 55% in advanced stages [7]. These findings highlight the need not only for early detection but also for reliable prognostic biomarkers to predict recurrence risk. Such biomarkers may contribute to improved molecular characterization and risk assessment while potentially helping to reduce unnecessary surgical interventions [8]. Furthermore, biomarker-guided approaches may contribute to the development of targeted therapeutic strategies and help minimize the risk of disease recurrence [7].

Currently, fine-needle aspiration biopsy (FNAB) is considered the gold standard for the evaluation of thyroid nodules; however, it yields indeterminate results in approximately 15–30% of cases [9, 10]. This situation creates a clinical dilemma between performing potentially unnecessary surgery and the risk of overlooking malignancy. In recent years, commercial molecular diagnostic tests such as Afirma Genomic Sequencing Classifier and ThyroSeq Genomic Classifier have been introduced to address this diagnostic uncertainty [9]. Nevertheless, their high costs limit accessibility and widespread use, particularly in developing countries. Beyond molecular testing, adjunctive computational approaches—including artificial intelligence–assisted analysis of cytological material—are increasingly being explored to improve the risk stratification of indeterminate nodules, reflecting the broader recognition that conventional cytology and currently available molecular tests alone may be insufficient for reliable classification [11]. Therefore, there is a clear need to develop novel biomarkers with high diagnostic accuracy and sensitivity that are also cost-effective, which could ultimately support the molecular characterization of thyroid malignancies and inform the future evaluation of indeterminate thyroid nodules [12].

Among the most frequently used molecular markers for thyroid cancer diagnosis are BRAF mutations, RAS mutations, RET/PTC rearrangements, and PAX8/PPARγ fusion genes [13–15]. In particular, the BRAF V600E mutation shows a strong association with PTC and has demonstrated a specificity of approximately 98–100% in several meta-analyses. However, its diagnostic sensitivity remains limited because the mutation is detected in only about 35–65% of PTC cases [8, 16, 17]. RAS mutations, on the other hand, occur more frequently in follicular thyroid carcinoma than in PTC. Moreover, because these mutations are observed in a wide range of other malignancies, their diagnostic value as a standalone marker is limited by relatively low sensitivity and specificity [18, 19]. RET/PTC rearrangements are detected in approximately 20% of sporadic PTC cases. Collectively, these limitations indicate that approaches based on a single genetic alteration are insufficient to serve as universal biomarkers for diagnosing PTC [20, 21]. Therefore, identifying novel biomarkers with higher diagnostic accuracy remains a priority for improving the early detection and clinical management of PTC.

Advances in high-throughput transcriptomic profiling technologies and systems biology approaches have enabled the identification of diagnostic and prognostic biomarkers across a wide range of malignancies using gene expression data [22]. In this context, Least Absolute Shrinkage and Selection Operator (LASSO) regression–based feature selection provides a robust and reproducible analytical framework for identifying the most discriminative gene subsets from high-dimensional datasets [23].

Several genes implicated in diverse biological processes may contribute to the molecular heterogeneity of PTC. These include genes involved in neurotransmitter signaling (e.g., *GABRB2* and *GRIA1*), membrane lipid metabolism (*SERINC2*), Hedgehog signaling regulation (*PTCHD4*), extracellular matrix organization (*HAPLN1*), amino acid transport (*SLC6A15*), and detoxification pathways (*UGT2B11*). Dysregulation of these pathways has been associated with tumor proliferation, invasion, metabolic adaptation, and tumor microenvironment remodeling across multiple cancer types [24–29]. Therefore, integrative transcriptomic approaches may facilitate the identification of gene signatures reflecting these diverse biological mechanisms and contribute to improved molecular characterization of PTC.

In this study, we aimed to identify a seven-gene signature that could improve the diagnostic and prognostic evaluation of PTC. To achieve this, transcriptomic data from The Cancer Genome Atlas (TCGA) thyroid carcinoma cohort (TCGA-THCA) were integrated with LASSO logistic regression to develop a seven-gene expression signature capable of distinguishing PTC from normal (tumor-adjacent) thyroid tissues with high accuracy. The proposed signature demonstrated excellent diagnostic performance in the discovery cohort, achieving an area under the curve (AUC) of 0.995, and was successfully validated in two independent Gene Expression Omnibus (GEO) datasets (GSE33630 and GSE60542). The prognostic value of this signature was further evaluated using disease-free interval (DFI), progression-free interval (PFI), and overall survival (OS) analyses. In addition, DNA methylation–expression correlation analyses were conducted to explore the potential epigenetic regulatory mechanisms of the signature genes, and single-sample gene set enrichment analysis (ssGSEA)–based immune cell infiltration analysis was performed to elucidate the relationship between the identified gene signature and the tumor microenvironment.

## Results

### Identification of differentially expressed genes in PTC

**Fig. 1:**
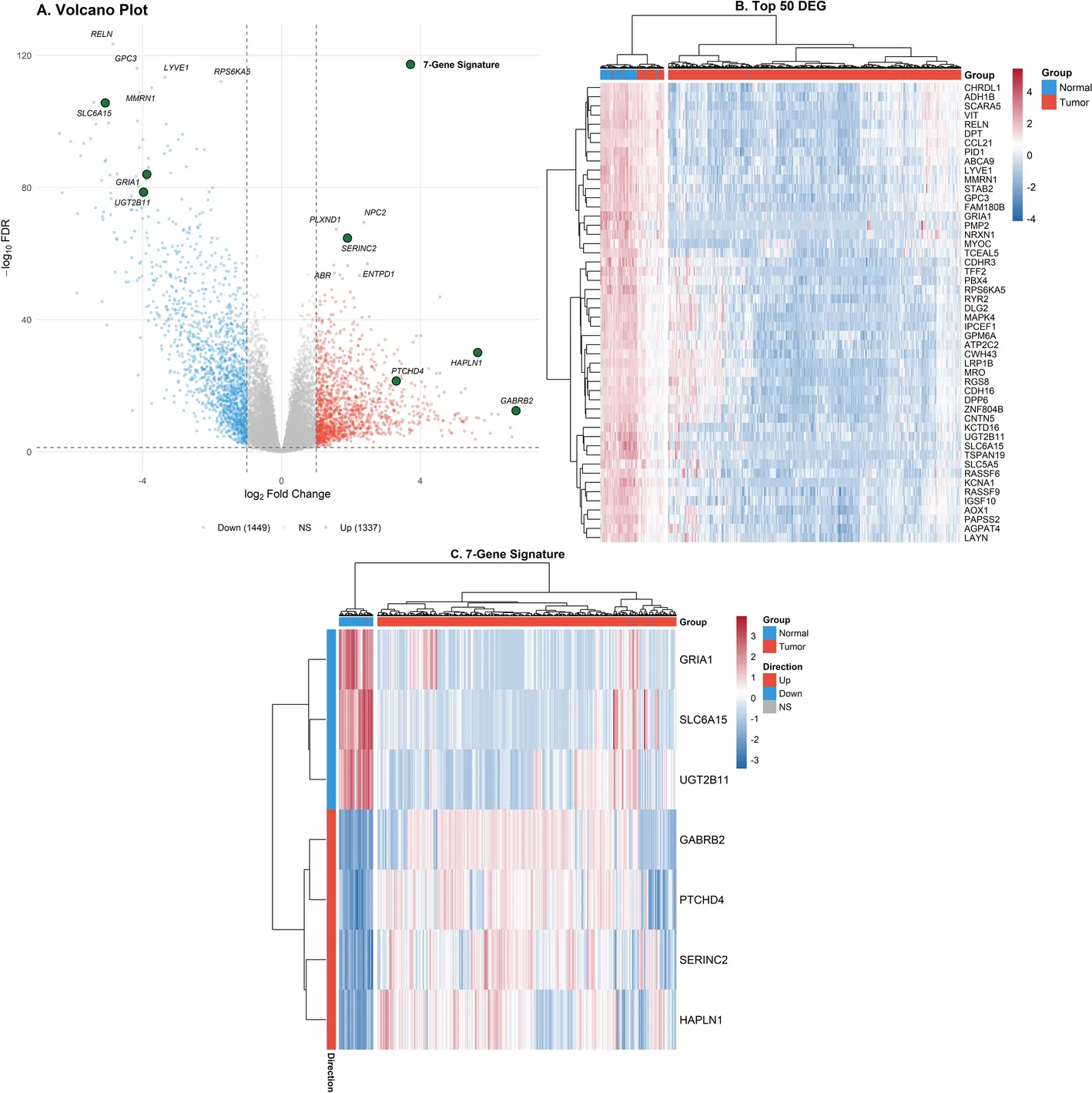
Differential gene expression analysis in PTC. **A** Volcano plot showing differentially expressed genes (DEGs) between tumor and normal thyroid tissues in the TCGA-THCA cohort (FDR < 0.05, |log₂FC|> 1). Red dots represent upregulated genes (n = 1337), blue dots downregulated genes (n = 1449), and gray dots non-significant genes. Green points highlight the seven genes included in the diagnostic signature. **B** Heatmap of the top 50 DEGs ranked by FDR, with hierarchical clustering of samples and genes. Red indicates higher expression and blue lower expression; the top annotation bar denotes normal (blue) and tumor (red) groups. **C** Heatmap of the seven signature genes across tumor and normal samples, annotated by group (Normal/Tumor) and regulation direction (Up/Down)

A total of 2786 differentially expressed genes (DEGs) were identified between PTC and normal thyroid tissues, including 1337 upregulated and 1449 downregulated genes. The distribution of DEGs is presented in the volcano plot (Fig. 1A), and the expression patterns of the top DEGs are shown in the heatmap (Fig. 1B). Several genes subsequently included in the diagnostic signature were among the significantly dysregulated genes. Expression profiles of the seven signature genes across tumor and normal samples are shown in Fig. 1C.

### Functional enrichment analysis of DEGs

**Fig. 2:**
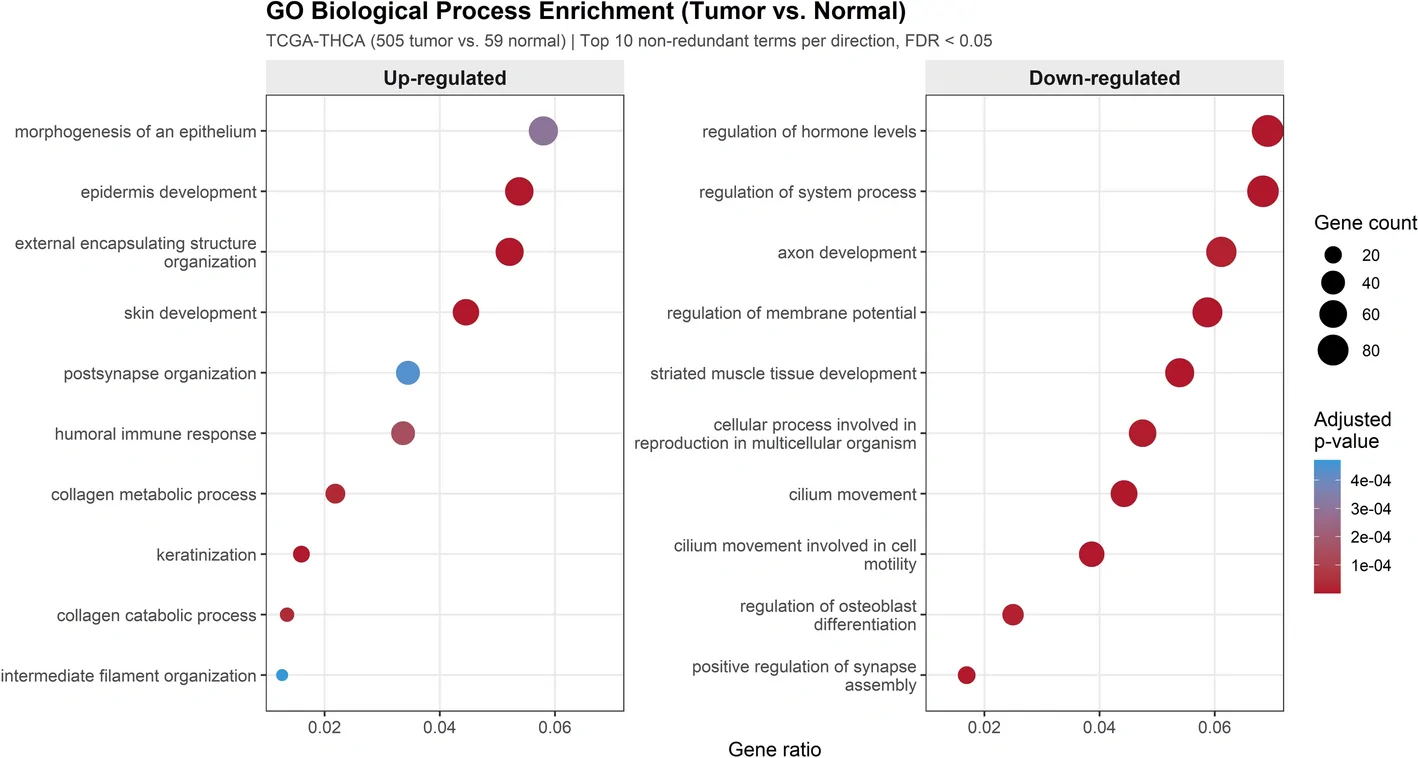
GO biological process enrichment analysis of DEGs in PTC. Dot plots showing the top 10 non-redundant Gene Ontology biological process terms enriched among upregulated (left) and downregulated (right) genes in tumor versus normal thyroid tissue (TCGA-THCA; 505 tumor vs. 59 normal; FDR < 0.05). Dot size represents gene count, and color intensity indicates the adjusted p-value (FDR). The x-axis represents the gene ratio

Gene Onthology (GO) biological process enrichment analysis demonstrated that upregulated genes were primarily associated with extracellular matrix organization, epidermis development, epithelial differentiation, and collagen-related processes, whereas downregulated genes were enriched in cilium movement, hormone-related processes, and regulation of membrane potential (Fig. 2).

KEGG pathway analysis further showed enrichment of several cancer-related pathways among upregulated genes, including ECM–receptor interaction, PI3K–Akt signaling, thyroid hormone signaling, cell cycle regulation, and chronic myeloid leukemia pathways. The p53 signaling pathway also showed nominal enrichment. In contrast, no KEGG pathway remained significant among downregulated genes after FDR correction, although several pathways demonstrated nominal enrichment prior to correction, including mineral absorption, hormone signaling, dopaminergic synapse, glutamatergic synapse, and adipocytokine signaling pathways.

### Development and diagnostic performance of the seven-gene signature

**Fig. 3:**
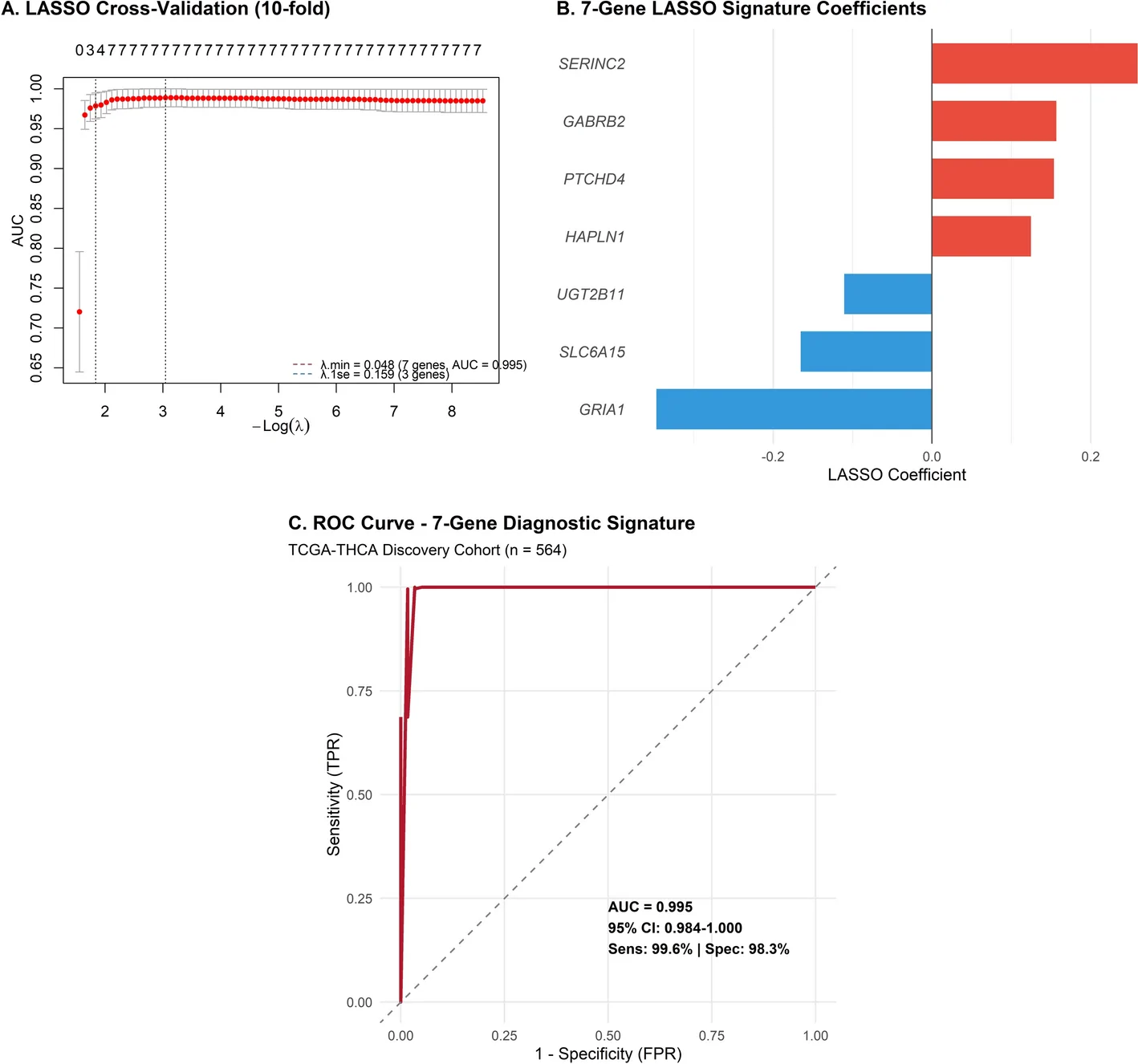
Development of the 7-gene diagnostic signature using LASSO logistic regression. **A** Ten-fold cross-validation curve for LASSO regularization. The y-axis shows mean AUC and the x-axis − log(λ); numbers above the plot indicate the number of selected genes. The left dashed line indicates λ.1se (3 genes) and the right dashed line indicates λ.min (0.048; 7 genes, AUC = 0.995). **B** LASSO coefficients of the seven selected genes; upregulated genes are shown in red and downregulated genes in blue. **C** Receiver operating characteristic (ROC) curve of the seven-gene signature in the TCGA-THCA discovery cohort (n = 564). AUC = 0.995 (95% CI: 0.984–1.000); sensitivity 99.6%, specificity 98.3%

LASSO logistic regression identified a seven-gene diagnostic signature comprising four upregulated genes (*GABRB2*, S*ERINC2*, *PTCHD4* and *HAPLN1*) and three downregulated genes (*GRIA1*, *SLC6A15* and *UGT2B11*) (Fig. 3A–B). The resulting model demonstrated excellent diagnostic performance in the TCGA-THCA discovery cohort, with an AUC of 0.995 (95% CI: 0.984–1.000), sensitivity of 99.6%, and specificity of 98.3% (Fig. 3C).

The coefficients and statistical characteristics of the seven genes included in the diagnostic signature are summarized in Table 1.

**Table 1:** Seven-gene LASSO diagnostic signature for PTC

Gene	Coefficient	Regulation	log_2_FC	FDR	AUC	FDR Rank	Wilcoxon p-value
	− 0.347	DOWN	− 3.88	9.3e-85	0.948	35	4.1e-31
*SERINC2*	0.259	UP	1.90	1.9e-65	0.968	93	4.7e-32
*SLC6A15*	− 0.165	DOWN	− 5.08	2.3e-106	0.960	8	4.1e-32
*GABRB2*	0.157	UP	6.75	3.3e-13	0.982	1695	9.0e-34
*PTCHD4*	0.154	UP	3.31	3.3e-22	0.958	1014	9.3e-31
	0.125	UP	5.65	8.3e-31	0.958	613	1.1e-30
*UGT2B11*	− 0.111	DOWN	− 3.98	2.3e-79	0.956	46	1.4e-30

**Fig. 4:**
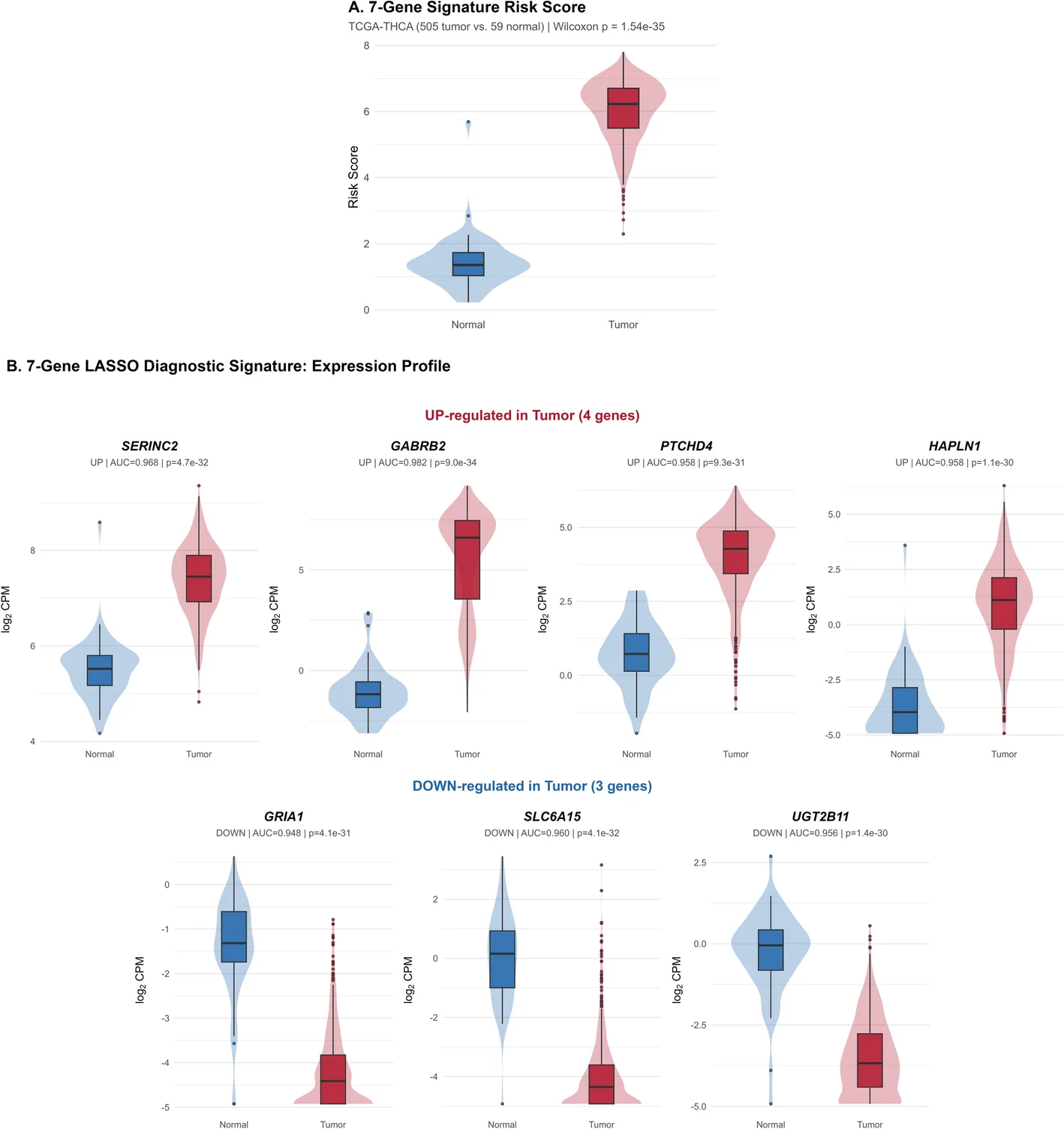
Risk score distribution and expression profiles of the 7-gene signature. **A** Violin-boxplot showing the distribution of risk scores between normal (n = 59) and tumor (n = 505) samples in the TCGA-THCA cohort (Wilcoxon rank-sum test, p = 1.54 × 10⁻^35^). **B** Expression profiles of the seven signature genes in normal versus tumor tissues, separated into genes upregulated in tumor (*SERINC2*, *GABRB2*, *PTCHD4*, *HAPLN1*) and downregulated in tumor (*GRIA1*, *SLC6A15*, *UGT2B11*). Each panel shows the log₂CPM distribution with the corresponding AUC and Wilcoxon p-value; all genes demonstrate high individual discriminatory ability (AUC > 0.94, all p < 10⁻^30^)

The risk score derived from the seven-gene signature showed a clear separation between tumor and normal samples (Fig. 4A). Consistent with the differential expression analysis, *SERINC2, GABRB2, PTCHD4,* and *HAPLN1* were upregulated in tumor tissues, whereas *GRIA1, SLC6A15, and UGT2B11* were downregulated (Fig. 4B).

### External validation of the seven-gene signature in ındependent GEO cohorts

**Fig. 5:**
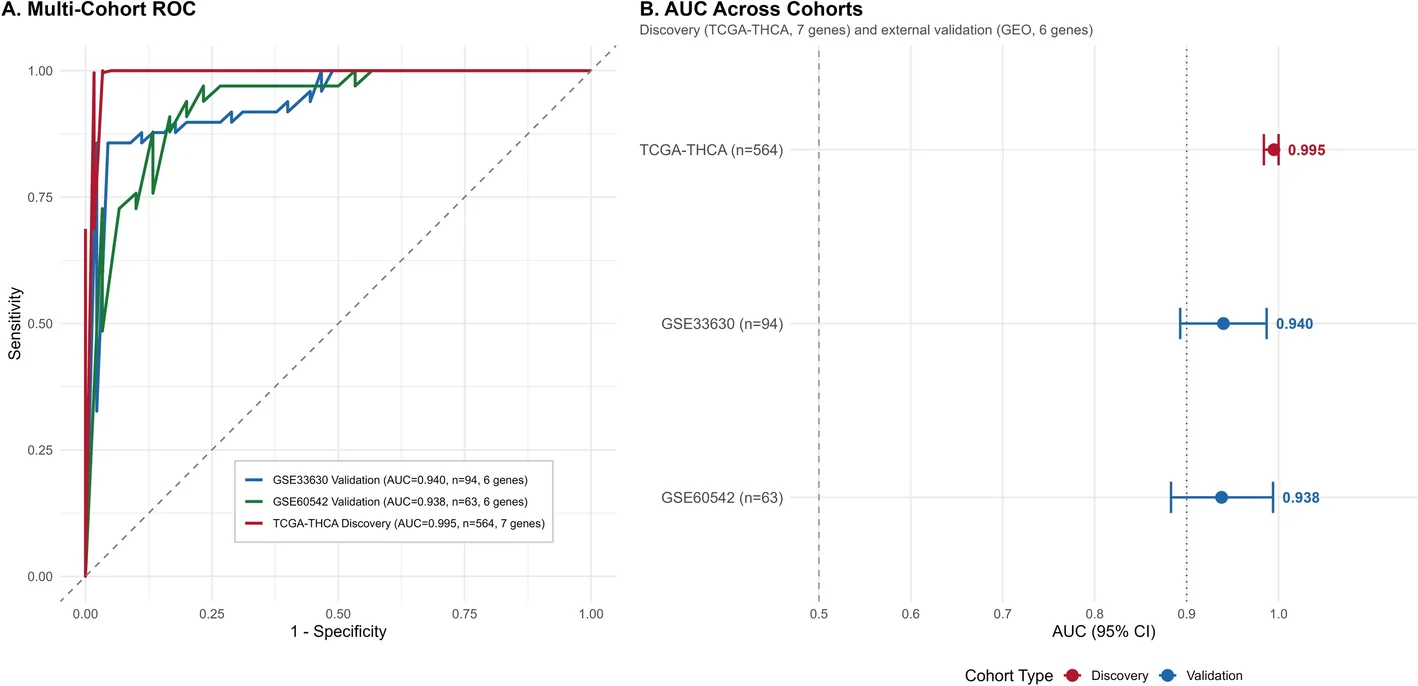
External validation of the seven-gene diagnostic signature across independent cohorts. **A** ROC curves for the TCGA-THCA discovery cohort (red; AUC = 0.995, n = 564, 7 genes) and the two independent GEO validation cohorts (GSE33630, blue, AUC = 0.940, n = 94, 6 genes; GSE60542, green, AUC = 0.938, n = 63, 6 genes). **B** Forest plot showing AUC values with 95% confidence intervals across cohorts (discovery in red, validation in blue). The dotted vertical line indicates an AUC threshold of 0.9

External validation confirmed the robustness of the seven-gene diagnostic signature across independent GEO cohorts (Fig. 5A–B). In the GSE33630 cohort (49 tumors and 45 normal samples), a six-gene model excluding *UGT2B11*achieved an AUC of 0.940 (95% CI: 0.893–0.987). Similarly, in the GSE60542 cohort (33 tumors and 30 normal samples), the six-gene model yielded an AUC of 0.938 (95% CI: 0.883–0.994). ROC curves for the discovery and validation cohorts are shown in Fig. 5A, whereas cohort-specific AUC estimates with 95% confidence intervals are summarized in Fig. 5B and Table 2.

**Table 2:** Diagnostic performance of the seven-gene signature across discovery and validation cohorts

Cohort	Type	N	AUC (95% CI)	Sensitivity (%)	Specificity (%)	Genes Used
TCGA-THCA	Discovery	564	0.995 (0.984–1.000)	99.6	98.3	7/7
GSE33630	Validation	94	0.940 (0.893–0.987)	85.7	97.8	6/7
GSE60542	Validation	63	0.938 (0.883–0.994)	87.9	86.7	6/7

Internal validation further demonstrated stable model performance. Repeated tenfold cross-validation in the TCGA-THCA cohort produced a cross-validated AUC of 0.983 (SD = 0.001), while an independent stratified hold-out test set yielded an AUC of 0.988 (95% CI: 0.971–1.000). Sensitivity analysis showed that exclusion of *UGT2B11* had minimal impact on diagnostic accuracy, with comparable performance observed between the seven-gene and six-gene models across all evaluation schemes.

### Prognostic value of the seven-gene signature

**Fig. 6:**
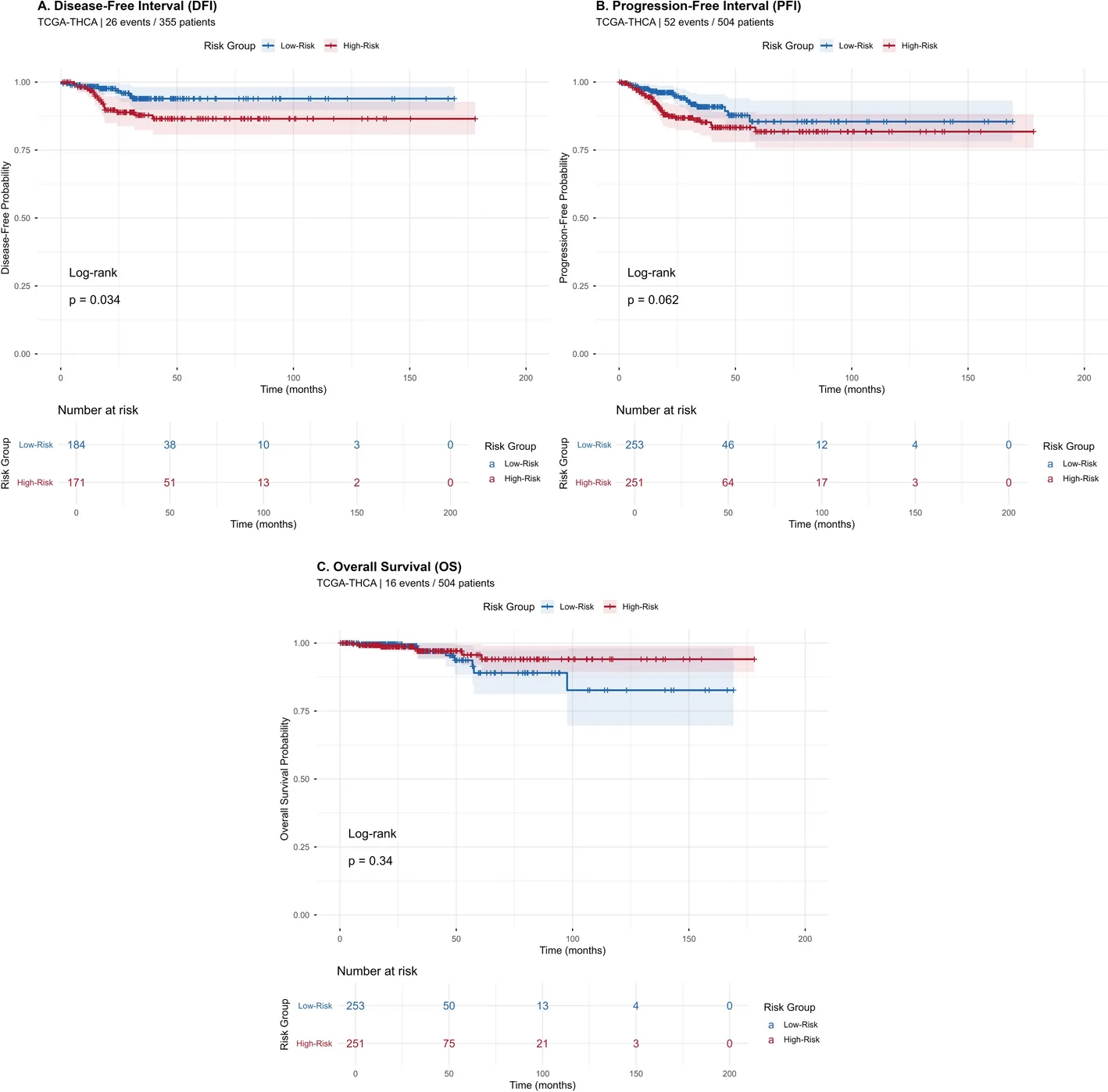
Prognostic evaluation of the seven-gene risk signature in the TCGA-THCA cohort. Patients were stratified into high-risk (red) and low-risk (blue) groups based on the median risk score, and survival was compared using the log-rank test. **A** Disease-free interval (DFI; 26 events / 355 patients; p = 0.034). **B** Progression-free interval (PFI; 52 events / 504 patients; p = 0.062). **C** Overall survival (OS; 16 events / 504 patients; p = 0.34). Shaded areas represent 95% confidence intervals, and number-at-risk tables are shown below each panel

Kaplan–Meier analysis demonsrated that patients in the high-risk group had a significantly shorter disease-free interval (DFI) than those in the low-risk group (log-rank p = 0.034; Fig. 6A). A similar trend was observed for progression-free interval (PFI), although the difference did not reach statistical significance (log-rank p = 0.062; Fig. 6B). In contrast, overall survival (OS) did not differ significantly between the two risk groups (log-rank *p* = 0.34; Fig. 6C).

**Fig. 7:**
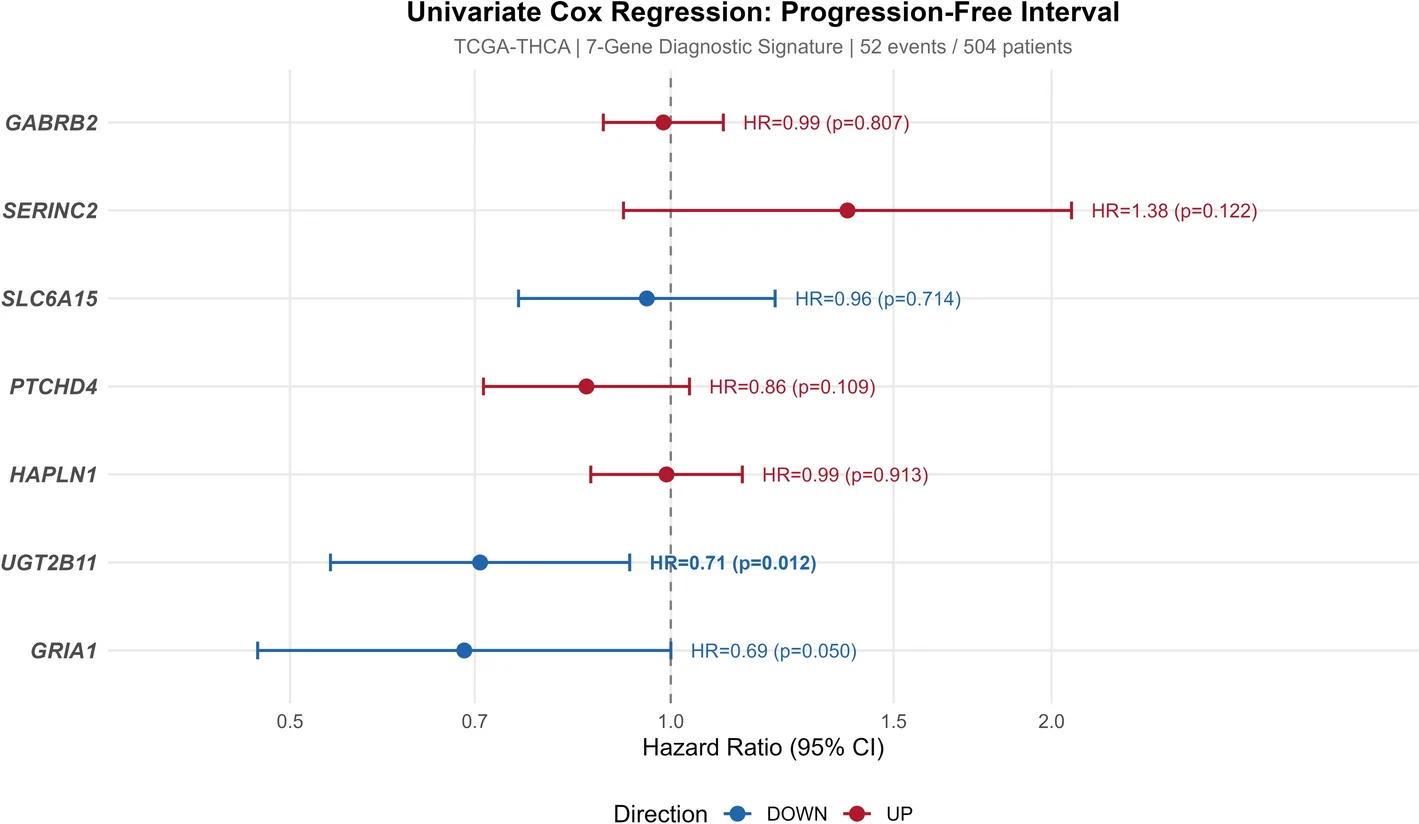
Univariate Cox regression analysis for PFI in the TCGA-THCA cohort. Forest plot showing hazard ratios (HR) and 95% confidence intervals for the seven signature genes (n = 504 patients, 52 events). *UGT2B11* (HR = 0.71, p = 0.012) was significantly associated with progression-free survival and *GRIA1* showed borderline significance (HR = 0.69, p = 0.050). The dashed vertical line represents HR = 1. Genes upregulated in tumors are shown in red and downregulated genes in blue

Univariate Cox regression analysis for PFI identified *UGT2B11* expression as significantly associated with progression-free survival (HR = 0.71, 95% CI: 0.54–0.93, *p* = 0.0139), whereas *GRIA1* showed borderline significance (HR = 0.69, *p* = 0.050) (Fig. 7). None of the remaining signature genes demonstrated significant associations with PFI.

Multivariable analysis showed that the risk score was not independently associated with either disease-free interval (DFI; adjusted HR = 1.40, 95% CI: 0.89–2.20, *p* = 0.14) or progression-free interval (PFI; adjusted HR = 1.09, 95% CI: 0.81–1.46, *p* = 0.56). In contrast, lymph node positivity was significantly associated with shorter PFI (HR = 1.96, 95% CI: 1.05–3.67, *p* = 0.035).

Overall survival analysis was underpowered because only 16 events were observed, limiting reliable assessment of an independent prognostic effect. The proportional-hazards assumption was satisfied in all models (global Schoenfeld residual test, *p* > 0.05).

### DNA methylation–expression correlation of signature genes

**Fig. 8:**
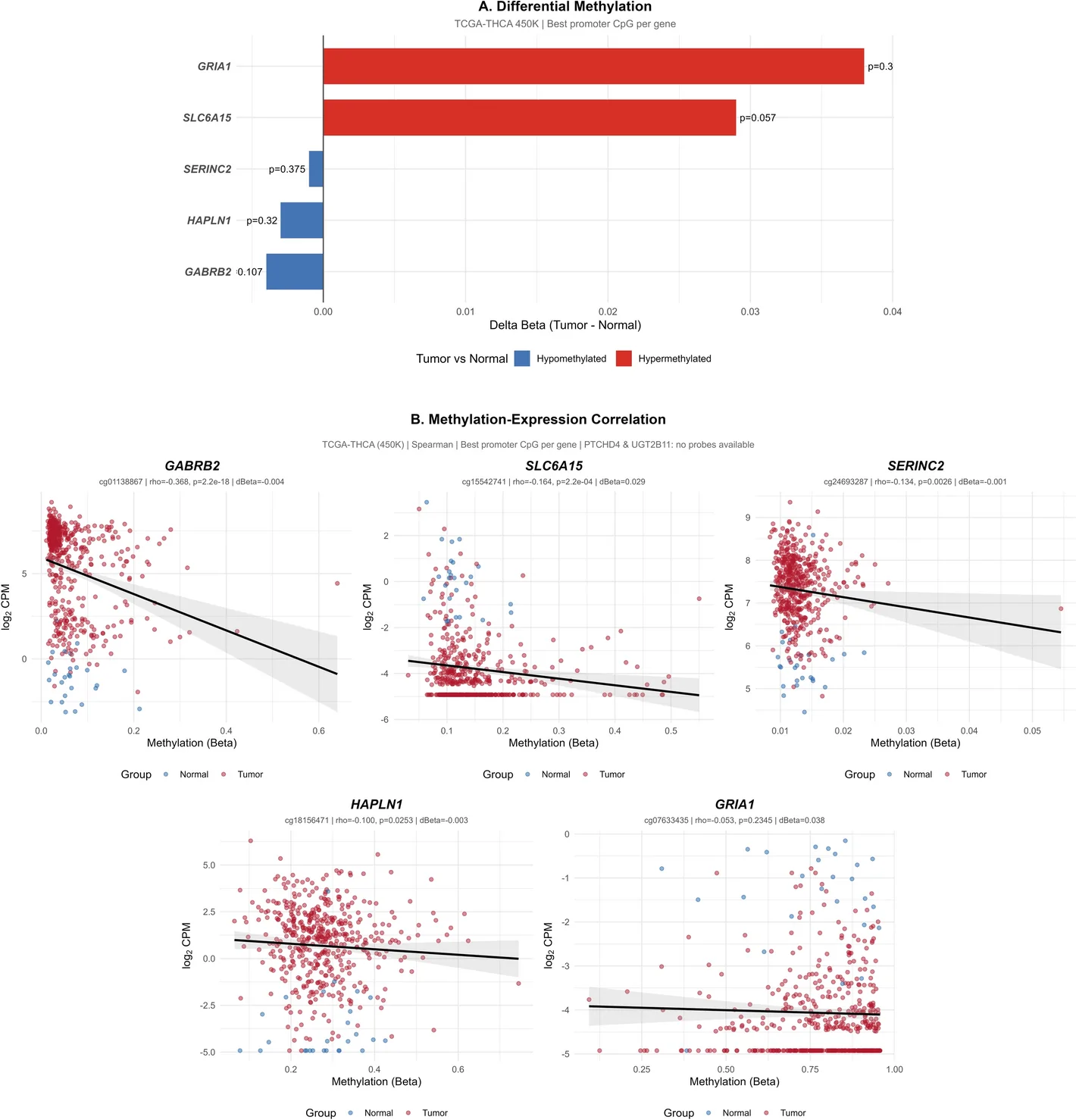
DNA methylation analysis of the signature. **A** Differential promoter methylation between tumor and normal tissues for the signature genes with annotated promoter CpG probes on the TCGA-THCA 450 K platform (best promoter CpG per gene). Delta Beta values (Tumor − Normal) are shown; red indicates hypermethylation and blue hypomethylation in tumor samples, with the corresponding p-value for each gene. **B** Methylation–expression correlation analysis (Spearman). Scatter plots show the relationship between promoter CpG methylation (Beta value) and gene expression (log₂CPM) in TCGA-THCA samples for *GABRB2*, *SLC6A15*, *SERINC2*, *HAPLN1*, and *GRIA1*; each panel reports the CpG probe ID, Spearman ρ, p-value, and Delta Beta. Tumor samples are shown in red and normal samples in blue, and black lines represent linear regression fits with 95% confidence intervals. No annotated promoter probes were available for *PTCHD4* and *UGT2B11*

DNA methylation–expression correlation analysis identified significant negative correlations between promoter methylation and gene expression for four signature genes: *GABRB2* (ρ = − 0.368, *p* = 2.2 × 10⁻^18^), *SLC6A15* (ρ = − 0.164, *p* = 2.2 × 10⁻^4^), *SERINC2* (ρ = − 0.134, *p* = 0.003), and *HAPLN1* (ρ = − 0.100, *p* = 0.025) (Fig. 8B). In contrast, *GRIA1* did not show a significant association between promoter methylation and gene expression (ρ = − 0.053, *p* = 0.235).

Differential methylation analysis demonstrated promoter hypermethylation of *GRIA1* and *SLC6A15*, whereas *GABRB2*, *HAPLN1*, and *SERINC2* showed relative hypomethylation in tumor tissues compared with normal thyroid tissue (Fig. 8A).

### Immune microenvironment differences between risk groups

**Fig. 9:**
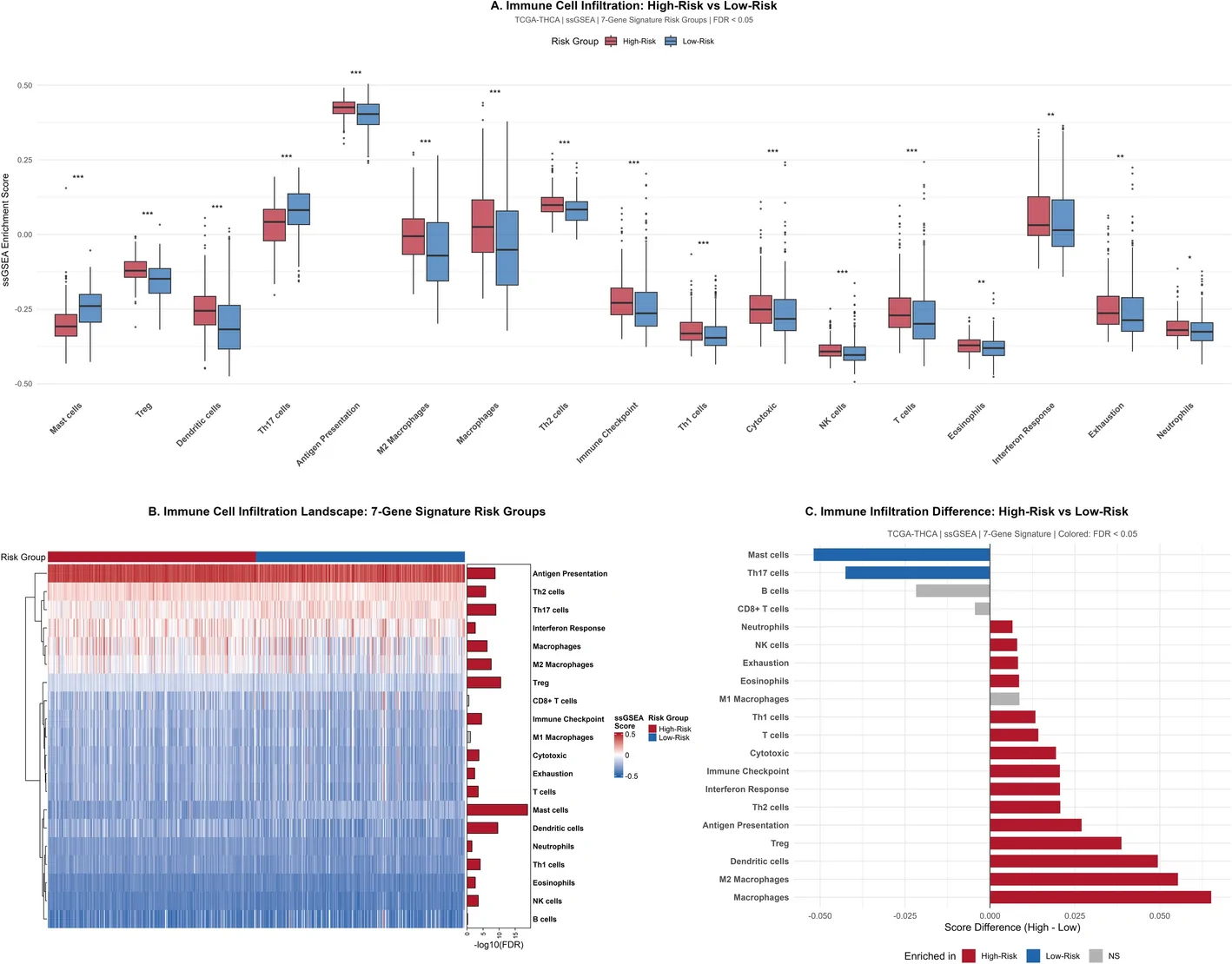
Immune cell infiltration analysis between risk groups defined by the seven-gene signature. **A** Boxplots comparing ssGSEA enrichment scores for 20 immune cell types and immune-related signatures between high-risk (red) and low-risk (blue) groups in TCGA-THCA. Statistical significance was assessed using the Wilcoxon rank-sum test with Benjamini–Hochberg correction (**FDR < *0.001,* *FDR < 0.01). **B** Heatmap of ssGSEA scores across TCGA-THCA tumor samples, with the top annotation indicating risk-group assignment; the adjacent bar plot shows the − log10(FDR) for each immune component. **C** Bar plot of mean score differences (high-risk minus low-risk) for each immune cell type, colored by enrichment direction (red, enriched in high-risk; blue, enriched in low-risk; gray, not significant)

Seventeen of the 20 immune components showed significant differences between the high-risk and low-risk groups (FDR < 0.05). The high-risk group demonstrated significantly higher enrichment scores for regulatory T cells (Treg; FDR = 2.9 × 10⁻^11^), dendritic cells (FDR = 2.5 × 10⁻^10^), antigen presentation signatures (FDR = 1.7 × 10⁻⁹), M2 macrophages (FDR = 2.8 × 10⁻⁸), total macrophage populations (FDR = 5.3 × 10⁻⁷), immune checkpoint signatures (FDR = 2.7 × 10⁻^5^), and exhaustion-related markers (FDR = 0.004) (Fig. 9A-C). In contrast, mast cells (FDR = 1.4 × 10⁻^19^) and Th17 cells (FDR = 9.8 × 10⁻^10^) were significantly reduced in the high-risk group.

## Discussion

PTC) is generally associated with a favorable prognosis; however, the risk of recurrence and diagnostic uncertainty remain important clinical challenges. According to global cancer statistics, thyroid cancer has increased from the 10th most common cancer in GLOBOCAN 2020 to the 7th in GLOBOCAN 2022, highlighting its growing epidemiological burden [30, 31]. Fine-needle aspiration biopsy (FNAB) remains the standard clinical method for evaluating thyroid nodules due to its practicality and reliability [10]. Nevertheless, indeterminate cytology occurs in approximately 15–30% of cases and may lead to either unnecessary surgery for benign nodules or delayed diagnosis of malignant lesions [9, 10]. Although commercial molecular diagnostic panels have been developed to address this diagnostic gap, their high cost and limited accessibility restrict widespread clinical use, particularly in resource-limited healthcare systems. Therefore, identifying reliable and cost-effective molecular biomarkers remains essential for improving the molecular characterization of thyroid malignancies.

Recent advances in systems biology approaches have facilitated the discovery of cancer biomarkers through network-based analyses. LASSO regression effectively selects the most informative variables from high-dimensional datasets while reducing overfitting, providing an efficient strategy for identifying disease-associated genes and molecular signatures [32, 33]. Previous studies have successfully applied LASSO-based feature selection to identify key genes associated with tumor progression and prognosis in various cancers, including hepatocellular carcinoma, lung squamous cell carcinoma, colorectal cancer, PTC, and stomach adenocarcinoma [32, 34–37].

In the present study, an integrative bioinformatics framework combining differential expression analysis (DEA) and LASSO logistic regression was applied to TCGA-THCA RNA-sequencing data integrated with two independent Gene Expression Omnibus (GEO) cohorts (GSE33630 and GSE60542), leading to the identification of a seven-gene diagnostic and prognostic signature for PTC. The proposed model demonstrated excellent diagnostic performance in the discovery cohort (AUC = 0.995; 95% CI: 0.984–1.000) and maintained strong predictive accuracy in independent validation datasets (GSE33630: AUC = 0.940; GSE60542: AUC = 0.938), indicating robust cross-platform generalizability. Furthermore, the signature showed a significant association with disease-free interval (DFI) in univariate Kaplan–Meier analysis; however, this association did not remain independent after multivariable adjustment for tumor stage and lymph node status, indicating that its prognostic relevance is, at present, hypothesis-generating.

To better understand the biological relevance of the identified signature, the functional roles of the seven genes were examined in the context of existing literature. Collectively, these genes represent multiple aspects of thyroid cancer biology and include four genes upregulated in tumor tissues (*GABRB2*, *SERINC2*, *PTCHD4*, and *HAPLN1*) and three genes downregulated in tumors (*SLC6A15*, *UGT2B11*, and *GRIA1*). The upregulated genes are associated with biological processes such as neuronal signaling, membrane lipid metabolism, Hedgehog pathway regulation, and extracellular matrix organization, suggesting that the signature captures diverse oncogenic mechanisms involved in tumor development and microenvironment remodeling. In contrast, the downregulated genes participate in normal physiological processes, including amino acid transport, detoxification metabolism, and glutamatergic signaling, indicating that disruption of these pathways may contribute to thyroid tumorigenesis.

Several genes included in our signature exhibit context-dependent roles across different cancer types. For example, *GABRB2* has been reported to show oncogenic characteristics in PTC [24], whereas *GRIA1*, which promotes tumor progression in glioblastoma [29], appears to exhibit a protective association in PTC. Similarly, *HAPLN1* has been implicated in tumor progression in gastric [26] and pancreatic cancers [38] but may function as a tumor suppressor in colorectal cancer [34]. *SERINC2* also demonstrates tissue-specific effects, showing oncogenic activity in lung cancer [25] and PTC [39] while displaying a potential protective role in clear cell renal cell carcinoma [40]. *SLC6A15* has been shown to act as a tumor suppressor in PTC by inhibiting cell migration and invasion via ICAM-1 [27], while its epigenetic silencing via DNA methylation has been reported in colorectal cancer [41, 42]. *UGT2B11* was the only one of the 12 highly expressed hepatic UGT genes consistently downregulated in hepatocellular carcinoma [28], and its reduced expression in PTC may indicate impaired detoxification capacity. Collectively, these findings further emphasize the heterogeneous and context-dependent roles of these genes across cancer types and support the importance of developing disease-specific molecular signatures and molecular subclassification approaches in cancer research [17, 47]. Several molecular biomarkers have been investigated for thyroid cancer diagnosis, including BRAF mutations, RAS mutations, RET/PTC rearrangements, and PAX8/PPARγ fusion genes [13–15]. Among these, the BRAF V600E mutation demonstrates high specificity but limited sensitivity because it is present in only a subset of PTC cases [16, 17]. Similarly, RAS mutations occur in multiple cancer types, reducing their specificity as standalone diagnostic markers [18, 19]. To address these limitations, commercial multi-gene panels such as the Afirma Genomic Sequencing Classifier [43] and the ThyroSeq Genomic Classifier have been developed to evaluate nodules with indeterminate cytology, while additional molecular profiling approaches using cytological thyroid specimens have also been explored in recent studies [44]. However, their high cost and limited accessibility restrict widespread implementation. In this context, the high specificity of the seven-gene signature observed in our study may support further investigation of its potential utility in molecular characterization strategies for PTC. However, this signature differs fundamentally from Afirma and ThyroSeq in sample type, clinical setting, and regulatory maturity: it was derived from bulk RNA-sequencing of tumor-versus-normal surgical tissue, whereas Afirma and ThyroSeq are analytically and clinically validated, commercially deployed assays intended specifically for fine-needle aspiration material from cytologically indeterminate nodules [5, 43]. The present signature has not undergone such analytical validation and was not tested on cytological or indeterminate-nodule samples; therefore, any role in reducing diagnostic surgery in indeterminate cytology remains a hypothesis to be tested directly in that setting rather than a demonstrated capability.

Although PTC generally exhibits excellent overall survival, recurrence occurs in up to 30% of patients [45]. Therefore, prognostic biomarkers should be evaluated not only for mortality but also for earlier clinical endpoints, such as disease recurrence and progression. In this study, the prognostic performance of the gene signature was evaluated using disease-free interval (DFI), progression-free interval (PFI), and overall survival (OS). The signature demonstrated a significant association with DFI, with high-risk patients exhibiting shorter disease-free intervals. Only borderline significance was observed for PFI, whereas no significant association was detected for OS—findings consistent with the generally favorable prognosis and low mortality rates characteristic of PTC. These prognostic associations were based on univariate analyses and a limited number of events. In multivariable models adjusted for tumor stage (and additionally for lymph node status in the PFI model), the risk score did not retain independent prognostic significance; accordingly, these associations should be regarded as hypothesis-generating rather than evidence of clinical prognostic utility.

Gene Ontology (GO) biological process analysis revealed that upregulated genes were significantly enriched in processes directly associated with tumor microenvironment remodeling and invasive potential, including extracellular matrix organization (FDR = 1.9 × 10⁻^10^), epidermal development (FDR = 4.2 × 10⁻⁹), collagen metabolism, and wound healing. Downregulated genes, in contrast, were enriched in processes reflecting normal thyroid tissue function, such as ciliary motility (FDR = 4.5 × 10⁻^11^), membrane potential regulation, and hormone metabolism. These findings are consistent with molecular alterations indicative of loss of differentiation in tumor tissue.

KEGG pathway analysis revealed comparable enrichment patterns. Upregulated genes were significantly enriched in pathways associated with established oncogenic mechanisms in thyroid carcinoma, including cornified envelope formation (FDR = 4.4 × 10⁻^4^)—a process linked to keratinocyte differentiation and keratinization—as well as cell cycle regulation (FDR = 0.002), chronic myeloid leukemia signaling (FDR = 0.034), RAS/MAPK signaling, and cell cycle checkpoint control. Among downregulated genes, pathways related to mineral absorption (FDR = 4.0 × 10⁻^4^) and hormone signaling (FDR = 0.005) were enriched at borderline statistical significance, consistent with impaired thyroid-specific functional activity in tumor tissue. Given the widespread transcriptional dysregulation observed, we next explored whether epigenetic mechanisms, particularly DNA methylation, may contribute to the regulation of signature genes.

DNA methylation–expression correlation analysis revealed significant negative correlations for several genes within the signature, most notably *GABRB2* (ρ = − 0.368, p = 2.2 × 10⁻^18^), supporting a potential role of epigenetic regulation in their transcriptional control. Although significant negative correlations were observed between methylation levels and gene expression, differential methylation analysis did not reveal statistically significant differences between tumor and normal tissues, suggesting that methylation variation within the tumor group, rather than a global shift, may drive these correlations. These findings suggest that transcriptional alterations may also be influenced by additional regulatory mechanisms such as transcription factors, chromatin remodeling, microRNA activity, or copy number variations. These methylation–expression relationships are correlational and do not establish causality; they should therefore be regarded as exploratory, hypothesis-generating observations rather than evidence of a defined epigenetic regulatory mechanism.

Beyond tumor-intrinsic gene expression alterations, the immune contexture of the tumor microenvironment was further examined to characterize the biological basis of the identified risk groups [46]. Immune infiltration analysis revealed significant differences in 17 of 20 immune cell types between high- and low-risk groups (FDR < 0.05). In particular, the enrichment of M2 macrophages and regulatory T cells in the high-risk group is consistent with an immunosuppressive tumor microenvironment that may facilitate tumor immune evasion and progression. In contrast, mast cells and Th17 cells were significantly enriched in the low-risk group, suggesting a more active anti-tumor immune response. Collectively, these findings suggest that the proposed gene signature captures not only tumor-intrinsic molecular alterations but also meaningful variation in the immune landscape of the PTC microenvironment. It should be emphasized that these ssGSEA-based immune estimates are computational inferences derived from bulk transcriptomic data and were not experimentally validated; the immune-microenvironment differences reported here are therefore exploratory and require confirmation by orthogonal methods such as immunohistochemistry or flow cytometry.

As a future direction, the compact size of the signature suggests that it could, in principle, be adapted to simpler and potentially more cost-effective assay formats (e.g., qPCR-based panels), which might be advantageous in resource-limited settings. We emphasize, however, that this is a prospective consideration rather than a current capability: the signature was developed and tested only on bulk transcriptomic data from surgical tissue, and any cost-effectiveness or practical assay performance would first need to be established through dedicated analytical and prospective clinical validation, including testing on fine-needle aspiration and indeterminate-nodule samples.

This study has several limitations that should be acknowledged. Most importantly, the present signature was developed and evaluated exclusively using bulk transcriptomic data from surgical tumor-versus-normal thyroid tissue; it was not tested on fine-needle aspiration or cytological material, nor on datasets containing benign nodules, follicular-patterned lesions, or cytologically indeterminate categories. Its diagnostic and risk-stratification performance in the clinically relevant setting of indeterminate thyroid nodules, therefore, remains to be established, and the present results should not be interpreted as evidence of immediate applicability to indeterminate cytology. In addition, the analyses were conducted using retrospective transcriptomic datasets derived from public databases. The biological roles of the identified genes were not experimentally validated, and in vitro or in vivo studies are warranted to confirm the proposed mechanistic links. The absence of UGT2B11 probes in the external validation datasets meant that a six-gene panel was applied during external validation. A sensitivity analysis in TCGA showed that the six-gene and full seven-gene models performed almost identically (cross-validated AUC 0.981 vs. 0.983; held-out test AUC 0.992 vs. 0.988, DeLong p = 0.36), indicating that the six-gene model is an adequate surrogate and that external validation is not materially compromised by the absence of UGT2B11. Nevertheless, formal evaluation of the complete seven-gene panel in an independent cohort with full gene coverage would still be valuable.

In addition, the discovery cohort was based on RNA-sequencing data from TCGA, whereas the validation cohorts were generated using microarray platforms. Although platform-specific normalization procedures were applied within each dataset, potential batch effects related to platform differences cannot be completely excluded. Fourth, the TCGA-THCA cohort predominantly represents North American populations, and the generalizability of the signature across diverse ethnic backgrounds requires further evaluation. Fifth, the low number of mortality events (n = 16) in the TCGA-THCA cohort limited the statistical power of the OS analysis. Nevertheless, the signature maintained high diagnostic accuracy across independent cohorts, supporting the robustness and generalizability of the identified risk stratification model.

In conclusion, this study identified and validated a novel seven-gene expression signature with strong diagnostic performance and potential prognostic relevance for PTC. The signature demonstrated excellent cross-platform robustness and showed associations with distinct immune infiltration patterns and exploratory DNA methylation alterations. Given the ongoing need for improved molecular characterization strategies in PTC, this multi-gene panel may represent a promising candidate for further investigation, pending validation in clinically relevant samples such as fine-needle aspiration and indeterminate-nodule specimens. Further experimental validation and prospective clinical studies are warranted to clarify its potential translational applicability.

## Methods

### Software and packages

All computational analyses were conducted using R software (version 4.3.2). The primary R packages used for data acquisition, preprocessing, statistical analyses, and visualization are summarized in Table 3.

**Table 3:** R packages and versions used in the present study

No.	R package	Version	Purpose
Data acquisition and preprocessing			
1	TCGAbiolinks	2.36.0	Downloading TCGA-THCA RNA-seq and clinical data from the GDC portal
2	GEOquery	2.76.0	Downloading GEO validation datasets (GSE33630, GSE60542)
Normalization and differential expression analysis			
3	edgeR	4.6.3	TMM normalization and differential gene expression analysis
4	Limma	3.64.3	Differential gene expression analysis using the voom/limma pipeline
Functional enrichment analysis			
5	clusterProfiler	4.16.0	Gene Ontology (GO) and KEGG pathway enrichment analysis
6	org.Hs.eg.db	3.21.0	Gene ID conversion and annotation database
7	Enrichplot	1.28.4	Visualization of enrichment analysis results
LASSO regression and diagnostic signature development			
8	Glmnet	4.1–10	LASSO regression for gene signature selection (tenfold cross-validation)
9	pROC	1.19.0.1	ROC curve analysis, AUC calculation, and confidence intervals
Prognostic and survival analysis			
10	Survival	3.8–3	Cox proportional hazards regression and survival analysis
11	Survminer	0.5.1	Kaplan–Meier survival curves and visualization
Immune infiltration analysis			
12	GSVA	2.2.0	Calculation of immune cell infiltration scores using ssGSEA
Visualization and data processing			
13	ComplexHeatmap	2.24.1	Visualization of immune infiltration heatmaps
14	Pheatmap	1.0.13	Gene expression heatmap visualization
15	ggplot2	4.0.2	General data visualization and Fig. generation
16	ggrepel	0.9.6	Non-overlapping gene labels in plots
17	VennDiagram	1.7.3	Visualization of DEG intersection Venn diagrams
18	RColorBrewer	1.1–3	Color palettes for graphical visualization
19	dplyr	1.1.4	Data manipulation and preprocessing

### Datasets and data sources

RNA-seq and DNA methylation data for thyroid carcinoma were obtained from The Cancer Genome Atlas (TCGA) database, while independent validation datasets were obtained from the Gene Expression Omnibus (GEO).

The details of the datasets used in this study are summarized in Table 4.

**Table 4:** Public datasets used in this study

Dataset	Platform	Data type	Source
TCGA-THCA	Illumina HiSeq RNA-Seq	Gene expression (RNA-seq)	https://portal.gdc.cancer.gov (accessed on December 30, 2025)
GSE33630	Affymetrix Human Genome U133 Plus 2.0	Gene expression (microarray)	https://www.ncbi.nlm.nih.gov/geo/query/acc.cgi?acc=GSE33630 (accessed on December 31, 2025)
GSE60542	Affymetrix Human Genome U133 Plus 2.0	Gene expression (microarray)	https://www.ncbi.nlm.nih.gov/geo/query/acc.cgi?acc=GSE60542 (accessed on December 31, 2025)
TCGA-THCA Methylation	Illumina HumanMethylation450 BeadChip	DNA methylation	https://xenabrowser.net

### Sample selection and cohort composition

Transcriptomic data (RNA-seq) for the thyroid carcinoma cohort were obtained from the TCGA database through the TCGAbiolinks R package. Data were downloaded from the Genomic Data Commons (GDC) portal using the current STAR-Counts workflow. The analysis included both primary tumor (n = 505) and normal thyroid tissue (n = 59) samples. Clinical data were retrieved using the GDCquery_clinic function and extracted from the colData slot of the SummarizedExperiment object.

For external validation, two independent microarray datasets (GSE33630 and GSE60542) were downloaded from the GEO. These datasets were generated using the Affymetrix Human Genome U133 Plus 2.0 platform and were processed in R using the GEOquery and limma packages. Because no annotated probe for the gene *UGT2B11* was available on this platform, external validation analyses were conducted using six of the seven genes included in the diagnostic signature.

DNA methylation profiles were obtained from the TCGA-THCA cohort using data generated by the Illumina Infinium HumanMethylation450 BeadChip platform. Beta values representing CpG methylation levels, ranging from 0 (unmethylated) to 1 (fully methylated), were downloaded from the UCSC Xena Browser. All datasets used in this study are publicly available, and therefore, ethical approval was not required.

### Data preprocessing and gene filtering

Raw RNA-seq count data were normalized using the trimmed mean of M-values (TMM) method implemented in the edgeR package in the R environment. Genes with low expression levels were filtered out, defined as those with counts per million (CPM) < 1 in more than 50% of the samples. The normalized expression values were subsequently transformed into log_2_ counts per million (log_2_-CPM) for downstream analyses.

Microarray datasets obtained from the GEO were processed using the GEOquery and limma packages in R. The raw microarray data were normalized using the Robust Multi-array Average (RMA) algorithm. Probe identifiers were mapped to gene symbols according to the corresponding platform annotation files. When multiple probes mapped to the same gene, the probe with the highest average expression value was retained for further analysis.

### Differential expression and functional enrichment analysis

#### Differential expression analysis

Differential expression analysis (DEA) between tumor and normal thyroid samples was performed using the quasi-likelihood framework implemented in the edgeR package in R. A generalized linear model (GLM) was applied to identify differentially expressed genes (DEGs). Genes with a false discovery rate (FDR) < 0.05 and an absolute log_2_ fold change (|log_2_FC|) > 1 were considered significantly differentially expressed. The distribution of DEGs was visualized using volcano plots and MA plots.

#### Functional enrichment analysis

To explore the biological mechanisms associated with the identified DEGs, Gene Ontology (GO) and KEGG pathway enrichment analyses were conducted using the clusterProfiler R package. Enrichment analyses were performed separately for upregulated and downregulated DEGs. Terms with an FDR-adjusted p-value < 0.05 were considered statistically significant.

All differentially expressed genes meeting the predefined thresholds (FDR < 0.05 and |log2FC|> 1; n = 2786) were used directly as the candidate gene set for LASSO feature selection. No additional pre-filtering (e.g., univariate effect-size or expression-based ranking) was applied before regularization; the L1 penalty itself performed the variable selection from the full DEG set.

#### LASSO logistic regression

To identify the optimal diagnostic gene subset, least absolute shrinkage and selection operator (LASSO) logistic regression was performed using the R package glmnet. The dependent variable was the sample class (tumor = 1, normal = 0), and the independent variables were the log_2_-CPM-normalized expression values of the candidate genes.

The optimal regularization parameter (λ) was determined using tenfold cross-validation, and the λ.min value corresponding to the highest area under the curve (AUC) was selected. Through L1 regularization, LASSO reduces model complexity by shrinking the coefficients of non-informative variables to zero, thereby addressing multicollinearity and generating a parsimonious predictive model.

#### Risk score calculation

For diagnostic analyses, samples were classified into high- and low-risk groups using the optimal cutoff value determined from the receiver operating characteristic (ROC) curve based on the Youden index (J = sensitivity + specificity − 1). For survival analyses, patients were stratified into high- and low-risk groups according to the median risk score.

The risk score for each sample was calculated as the linear combination of the LASSO regression coefficients and the log2-CPM-normalized expression values of the seven signature genes. Risk Score = ∑ (βᵢ × Exprᵢ), where βᵢ is the LASSO regression coefficient of gene i, and Exprᵢ is its log2-CPM-normalized expression value in the seven-gene signature.

### Diagnostic performance evaluation

#### ROC analysis

To evaluate the diagnostic performance of the identified gene signature, Receiver Operating Characteristic (ROC) curves were generated using the pROC package in R. The AUC and the corresponding 95% confidence intervals (CI) were calculated using the DeLong method. The discriminatory ability of each individual signature gene was also assessed by computing gene-level ROC curves and AUC values from the log2-CPM-normalized expression values, using the same pROC framework.

#### External validation

The robustness of the identified gene signature was further evaluated using independent validation datasets obtained from the GEO. In each validation cohort, risk scores were computed using exactly the same formula and the same LASSO regression coefficients derived from the TCGA-THCA discovery cohort (restricted to the six genes available on the microarray platforms), with no re-fitting or re-estimation of coefficients, and ROC analyses were performed to determine the corresponding AUC values. In addition to external validation, the internal validity of the discovery model was assessed in two complementary ways. First, repeated tenfold cross-validation (50 repeats) was performed in TCGA-THCA, in which the diagnostic model was refit on the training folds at each split and used to generate out-of-fold predictions that were pooled before computing the AUC. Second, the discovery cohort was randomly partitioned into a stratified 70% training and 30% testing set, and the model fit on the training set was evaluated on the held-out test set. To quantify the dependence of diagnostic performance on UGT2B11, whose probe was unavailable on the GEO microarray platforms, a sensitivity analysis compared the full seven-gene model with a six-gene model excluding UGT2B11, using apparent, cross-validated, and held-out AUCs together with the DeLong test for paired ROC curves.

### Survival analysis

#### Survival endpoints

Survival endpoints were defined according to the TCGA Pan-Cancer Clinical Data Resource (TCGA-CDR) established by Liu et al. [47]. Overall survival (OS) was defined as the time from the initial diagnosis to death from any cause. Disease-free interval (DFI) was defined as the time from the initial diagnosis to disease recurrence, whereas progression-free interval (PFI) was defined as the time from the initial diagnosis to disease progression or recurrence.

#### Kaplan–Meier analysis

Kaplan–Meier survival analyses for OS, DFI, and PFI were performed using clinical data from TCGA-THCA tumor samples. Patients were stratified into high- and low-risk groups based on the median risk score derived from the seven-gene signature. Kaplan–Meier survival curves were generated using the survival and survminer R packages. Differences in survival between groups were assessed using the log-rank test.

#### Cox regression analysis

To evaluate the association between the risk score and survival outcomes, univariate Cox proportional hazards regression analysis was performed, treating the risk score as a continuous variable. Hazard ratios (HRs) and corresponding 95% confidence intervals (CIs) were calculated. Subsequently, multivariable Cox regression was performed to assess whether the risk score (modeled as a continuous, standardized variable) remained an independent prognostic factor after adjustment for relevant clinicopathological variables. Candidate covariates considered included age, sex, tumor stage, and lymph node status, as well as BRAF and TERT status where available; however, given the limited number of events for each survival endpoint, the number of covariates was deliberately restricted to maintain an adequate events-per-variable ratio and to avoid model overfitting. The multivariable models were therefore adjusted for tumor stage (early [I/II] vs. advanced [III/IV]), with the progression-free interval model additionally adjusted for lymph node status (N0 vs. N +). The proportional-hazards assumption was evaluated using scaled Schoenfeld residuals. In addition, univariate Cox proportional hazards regression was performed separately for each of the seven signature genes, modeling the log2-CPM-normalized expression of each gene as a continuous variable, to evaluate its individual association with the progression-free interval; hazard ratios and 95% confidence intervals were estimated for each gene.

#### Individual gene survival analysis

Kaplan–Meier survival analyses were also performed for each gene included in the signature. Patients were divided into high- and low-expression groups based on the median expression value for each gene, and survival differences between groups were evaluated using the log-rank test.

### DNA methylation analysis

#### Methylation data processing

DNA methylation data generated using the Illumina HumanMethylation450 BeadChip platform were annotated using the IlluminaHumanMethylation450kanno.ilmn12.hg19 annotation package in R. Beta values, representing the ratio of methylated probe intensity to the total probe intensity, were used for downstream analyses. For each gene included in the signature, CpG probes located in promoter-related regions (TSS200, TSS1500, 1st exon, and 5′ UTR) were specifically selected.

#### Differential methylation analysis

Differentially methylated CpG sites between tumor and normal samples were identified using the Wilcoxon rank-sum test. P-values were adjusted for multiple testing using the Benjamini–Hochberg FDR method. CpG sites with an absolute methylation difference (|Δβ|> 0.05) and FDR < 0.05 were considered significantly differentially methylated.

#### Expression–methylation correlation analysis

Spearman's rank correlation analysis was performed to evaluate the relationship between DNA methylation and gene expression levels. Samples with matched RNA-seq and DNA methylation data were identified using patient barcodes. For each gene, the mean promoter methylation level (average β value across promoter-associated CpG sites) was correlated with normalized gene expression values (log_2_-CPM) to investigate the potential epigenetic regulation of gene expression.

#### Immune cell ınfiltration analysis

The composition of immune cells within the tumor microenvironment was evaluated using the single-sample gene set enrichment analysis (ssGSEA) method implemented in the GSVA R package. Gene signatures representing major immune cell populations—including B cells, T cells, CD8⁺ T cells, Th1, Th2, Th17, regulatory T cells (Treg), natural killer (NK) cells, dendritic cells, mast cells, neutrophils, and eosinophils—were obtained from the study by Bindea et al. [48].

Additional immune-related functional signatures, including macrophage polarization (M1/M2), cytotoxic activity, immune exhaustion, immune checkpoint signaling, antigen presentation, and interferon response pathways, were compiled from previous studies [46, 49].

In total, 20 immune cell types and functional immune signatures were evaluated. Patients were stratified into high- and low-risk groups based on the median risk score from the seven-gene signature. Differences in ssGSEA enrichment scores between groups were compared using the Wilcoxon rank-sum test, and p-values were adjusted using the Benjamini–Hochberg method. FDR < 0.05 was considered statistically significant.

### Statistical analysis

All statistical analyses were performed using R software. Comparisons of continuous variables between groups were conducted using the Student’s *t*-test for normally distributed data or the Wilcoxon rank-sum test for non-normally distributed data. Categorical variables were compared using the chi-square test or Fisher’s exact test, as appropriate.

For multiple comparisons, p-values were adjusted using the Benjamini–Hochberg method to control the FDR. All statistical tests were two-tailed, and a p-value < 0.05 was considered statistically significant.

## Data Availability

All datasets analyzed in this study are publicly available. The TCGA-THCA RNA-seq and DNA methylation datasets were obtained from the Genomic Data Commons (GDC) Data Portal (https://portal.gdc.cancer.gov/projects/TCGA-THCA; Accession: TCGA-THCA). The GSE33630 dataset is available at (https://www.ncbi.nlm.nih.gov/geo/query/acc.cgi?acc=GSE33630; Accession: GSE33630). The GSE60542 dataset is available at (https://www.ncbi.nlm.nih.gov/geo/query/acc.cgi?acc=GSE60542; Accession: GSE60542). DNA methylation data were accessed via the UCSC Xena Browser (https://xenabrowser.net/datapages/).
